# Is the Sustainable Choice a Healthy Choice?—Water Footprint Consequence of Changing Dietary Patterns

**DOI:** 10.3390/nu12092578

**Published:** 2020-08-25

**Authors:** Orsolya Tompa, Zoltán Lakner, Judit Oláh, József Popp, Anna Kiss

**Affiliations:** 1 Department of Food Chain Management, Institute of Agribusiness, Faculty of Economics and Social Sciences, Szent István University, 2100 Gödöllő, Hungary; orsolya.tompa@olympian.org (O.T.); lakner.zoltan@etk.szie.hu (Z.L.); 2 Faculty of Economics and Business, University of Debrecen, 4032 Debrecen, Hungary; 3 TRADE Research Entity, North-West University, Faculty of Economic and Management Sciences, Vanderbijlpark 1900, South Africa; popp.jozsef@szie.hu; 4 Faculty of Economics and Social Sciences, Szent István University, 2100 Gödöllő, Hungary; 5 Institute of Health Promotion and Sport Sciences, Faculty of Education and Psychology, Eötvös Loránd University, 1117 Budapest, Hungary; anna.kiss@pro-sharp.hu; 6 Department Science Policy and Scientometrics, Library and Information Centre of the Hungarian Academy of Sciences, 1051 Budapest, Hungary

**Keywords:** dietary scenarios, water footprint, sustainable nutrition, comparative analysis

## Abstract

It is evident that the modification of dietary patterns is a necessary precondition of disease prevention and health improvement. Changing nutritional habits also has deep-rooted consequences on the environmental burden. The majority of similar previous studies have analyzed the change in greenhouse gas emissions against theoretical modifications in current food consumption. The analysis on the effect of diet on the water footprint is also gaining in importance, since water supply is a critical global issue. Based on current nutritional patterns of a Central European country—Hungary—as well as dietary recommendations and scientific literature, we generated six dietary scenarios and determined the consequences of these on green (originally from precipitation) and blue (sourced from surface or groundwater) water consumption and dietary quality. Compared to the baseline scenario (current local nutritional pattern) of both genders, based on the integrated aspect of water footprint and dietary quality, the most disadvantageous scenario was the ketogenic (ca. −2% in dietary quality, +18% in blue water footprint, and +16% in green water footprint) and the most advantageous was the sustainable scenario (ca. +9% in dietary quality, −42% in green water footprint, and −29% in blue water footprint). As a summary it can be stated, that (1) there is no clear linear relationship between the “healthiness” and water footprint of different diets, but (2) a more balanced diet, which integrates nutritional and environmental considerations could decrease the environmental burden in an efficient way.

## 1. Introduction

The food industry is an important and dynamically evolving sector of the EU economy and every member country. It also participates in the fulfilment process of basic human needs [1,2,3]. At the same time, it is well documented that food quality considerably influences the health condition of a given population [4]. Moreover, it has become increasingly evident that the environmental consequences of changes in nutritional habits must be taken into consideration, too. Nowadays, one of the most challenging and urgent issues for humanity is to ensure a sustainable future, including a sustainable food system. The food system is at a risk due to the rapidly growing world population, climate change and the depletion of our natural resources such as arable land and fresh water [5,6]. On the other hand, food production is responsible for 70% of the total anthropogenic water use and it is the major source of water pollution [5]. Sustainable nutrition is an approach to ensure the sustainability of the food system; it includes different aspects and unites them as a whole [7,8]. In the opinion of Ingram et al. [9], the sustainability of nutrition can be evaluated in a seven-dimensional space. These dimensions are as follows: Food nutrient adequacy, ecosystem stability, food affordability and availability, sociocultural well-being, resilience, food safety, and waste and loss reduction. Ecosystem stability can be evaluated by different measures such as greenhouse gas emissions (GHGE), land use, water use, chemical emissions, and loss of biodiversity [10].

Previous studies have found an association between dietary quality and environmental impact: A dietary shift toward plant-based diets would result in a lower environmental impact [11,12,13,14]; however, a shift toward an extreme change such as a vegan diet can not be a realistic aim for the near future. A vegan diet would result in a great shift from the recent nutrition but would not satisfy the “cultural acceptability” dimension of sustainable nutrition. Furthermore, in the case of the vegan diet, there is a high risk for micronutrient intake deficiency. Also, with the decrease of meat and animal-based food intake, there is always a risk of micronutrient deficiency [15], although this can be avoided by a well-balanced diet [16]. At the same time, one cannot forget that a dynamic growth of the supplements’ market on a global scale can be observed [17]. As we have seen, the evaluation of the environmental consequences of diet is a highly complex problem. From this set of environmental aspects, the current article analyzes the problems of the water footprint, because a more detailed analysis of a sole environmental impact factor shows a somewhat more controversial picture, but also provides deep-rooted results for the development of dietary guidelines involving sustainability (Table 1).

From among all these aspects of sustainable nutrition, the present study concentrated on the association of food nutrient adequacy (i.e., dietary quality) and an environmental aspect (i.e., water footprint) in the context of the Hungarian population. Our aim was to make an integrated and specific assessment of the association of food consumption, dietary quality, and water footprint. The recent trend in studies on sustainable nutrition is directed toward an urgent change in the dietary recommendation that respects the environment, as has already been carried out by the most advanced countries [7,8,26,27] and as is recommended by the Food and Agriculture Organization of the United Nations (FAO). The aim of the current study was to analyze the consequences of the current, and some recommended, dietary patterns on the water footprint, applying a small, lesser-known Central European, landlocked country, Hungary, as a value-added for a comparison of the environmental effects of different diets.

The academic value-added content of this study is as follows:

(1) Despite the majority of similar studies focusing on GHGE or several environmental factors, the present one focused on the water footprint and gives a more detailed analysis of that critically important issue.
(2) The studies on sustainable nutrition focus on countries and regions. There is a wide-ranging consensus in the literature [28] that sociocultural traditions and differences in food availability limit the application of general solutions, which is why each research study, focusing on the environmental consequences of changing dietary patterns in a given region or country, could be considered a contribution to a better understanding of the interplay between environment and nutrition. Since all dimensions related to sustainable nutrition (such as the economy, food availability, food safety, sociocultural aspects, traditional meals, health condition of the population, etc.) are regionally different, each study that focuses on a new region necessarily involves methodological novelty. The challenge of these analyses is to collect and compile data from different databases and to develop scenarios and their measurements to evaluate dietary quality, environmental impact, and the association between them [29]. According to our best knowledge, this is the first study to attempt to integrate healthiness and the environmental aspect in terms of the nutrition of Hungary; thus, the methods can be regarded as novelties. These methods were based on a critical literature review but needed to be innovatively applied in the context of Hungary. Based on these, this study can be regarded as a methodological novelty and its results can contribute to the development of dietary recommendations involving sustainable nutrition in Hungary.

## 2. Materials and Methods

**Figure 1:**
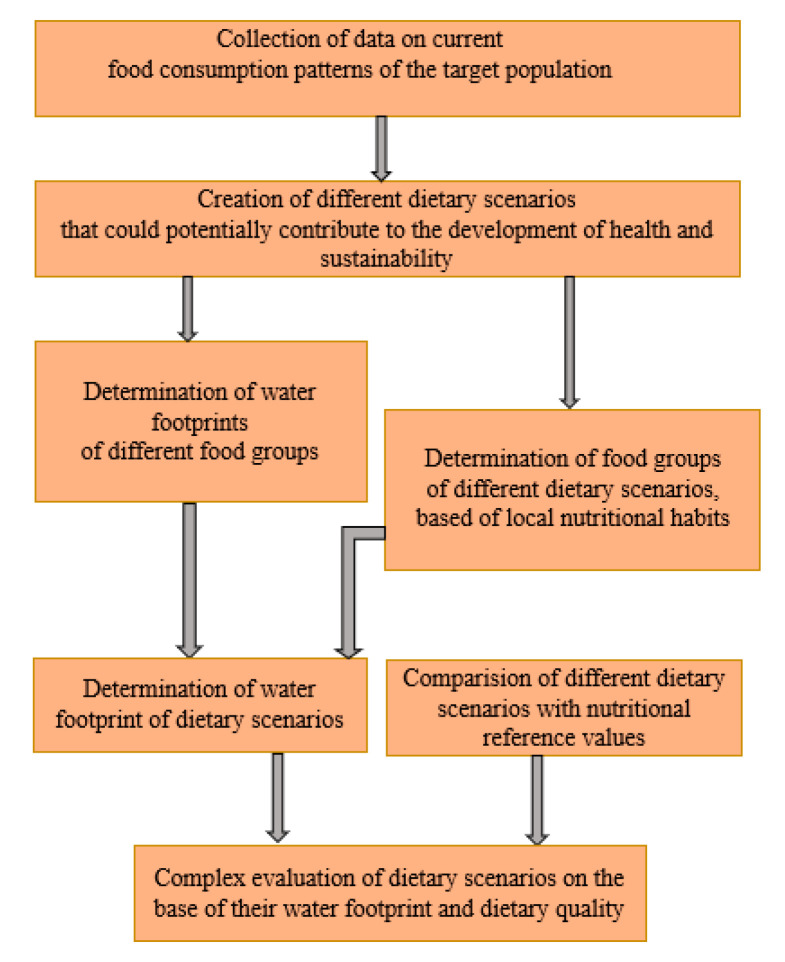
Logical framework of investigations.

In order to create an integrated analysis of sustainable nutrition in the context of the model country (Hungary), the authors estimated the food consumption pattern representing the current nutrition in Hungary as baseline scenario. The authors also created alternative scenarios based on dietary guidelines (sustainable and cardioprotective diet) and the description of an alternative diet (low-carbohydrate, high-fat diet) (Table 2.). These dietary scenarios refer to the whole adult and healthy population. They describe a particular version of possible future scenarios, so we could analyze their water footprint and dietary quality and the associations between them. As in the case of similar studies, besides sophisticated data collection and compilations, we applied relatively simple mathematics, as this seemed to be necessary and sufficient to fulfil the research goals. These types of studies are based on data collection, compilation, mathematical calculation, and analysis [10]. The topic in its current stage did not lends itself to the application of more detailed statistical apparatus. However, simple regression analysis between dietary quality values and water footprints separately was applied in the cases of both genders and water footprint types. For this statistical analysis, we considered 0.95 significance as a level of acceptance and we used IBM SPSS 22 software. The logical framework of our work is summarized in Figure 1.

### 2.1. Estimation of Food Consumption and the Proportion of Food Groups that Represent Current Nutrition in Hungary

**Figure 2:**
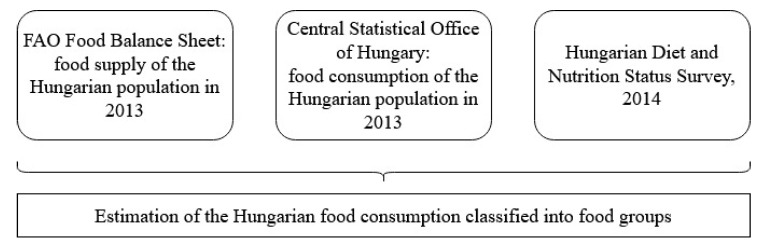
Estimation of the current food consumption in Hungary.

To estimate the current food consumption on a national level, there are different types of data sources that can be used. Most of the studies [22,23,30] rely on the FAO Food Balance Sheet (FBS) [31]), which contains data on the supply of food items within a country in different units. However, food supply is well accepted as a proxy of food consumption so it can correctly be used with correction factors, as described in the work of Vanham [30], since the FAO FBS contains data on unprocessed food items that are not equal to the prepared and consumed food items in measured units. In this present study, the data of the FAO FBS on food supply were used as a list of the most consumed food items and as weight factors that are equivalent to the amount of supply (Supplement 1). Supply units were transformed from kg/year/capita to g/day/capita as this is the reasonable amount in the context of daily food intake. Only food items that had a supply ≥4 g/day/capita were listed to ensure the inclusion of relevant food items. The data of the Central Statistical Office of Hungary, Budapest (CSO) [32] were also used to specify some food supply categories of the FAO FBS such as “Fruits, Other” in order to ensure that only fruits typical of the Hungarian consumption were included in the analysis (Supplement 1). The estimation of the proportional intake of different food groups (Figure 2) was based on the published results of The Hungarian Diet and Nutritional Status Survey (HDNSS), which was carried out in 2014 [33,34,35]. This survey is considered to be representative of the Hungarian population [33] and is based on analyzed dietary records that described the intake of different food groups in kcal/day/capita.

The nutrient density values of different food groups were calculated as the weighted averages of food densities of elements of the group. These latter data were acquired from the FoodData Central Database of the United States Department of Agriculture (USDA) [36], and the weights were the daily supply values.

(1) $$ND_{p}=\frac{\sum_{i=1}^{i=n}FI_{Si}FD_{i}}{\sum_{i=1}^{i=n}FI_{Si}}$$

where:

*ND_p_* = weighted average of the nutrient density of the pth food group (nutrient quantity/100 g),
*FI_s_* = supply of *ith* food item (100 g/day/capita), and
*FD_i_* = nutrient density of food item (nutrient quantity/100 g).

### 2.2. Characteristics of the Scenarios

The food consumption patterns of a given population can be approximated by analyzing average food consumption. To compare the effects of changes in the status quo, we had to define different possible modifications in the food consumption structure. A set of food consumption parameter values (practically: The food consumption structure) was termed a scenario. The current average dietary pattern was termed the status quo or baseline scenario. Other scenarios were defined on the basis of recommendations from international organizations and on the basis of a critical analysis of the literature. In the present study, the scenarios were based on the current nutrition of the Hungarian population, and reduced meat content, vegetarian, and vegan diets were also included. Besides these diets, scenarios based on sustainable, ketogenic, and cardioprotective diets were also included. A sustainable diet was included in the analysis, since it is the latest, environmentally conscientious approach in nutrition. A ketogenic diet was included because it is one of the most popular alternative diets; however, its high environmental impact is rarely considered [37]. A cardioprotective diet was also included, since it is the most relevant in the case of public health, since cardiovascular diseases are responsible for the greatest proportion of mortality rates in developed countries, as well as in Hungary (Institute for Health Metrics and Evaluation, Washington State, Seattle (IHME) [38]. A cardioprotective diet was already analyzed in terms of sustainability and showed promising results [25]. The proportion of the different food groups in kcal in each scenario is illustrated in Supplement 2. The characteristics of the different dietary scenarios are described in Table 2.

There are common characteristics for all dietary scenarios, as follows:

(1) All dietary scenarios are composed of food groups that include the weighted average of the most commonly consumed food items in Hungary in order to represent a relevant and culturally acceptable diet as close as possible to reality.
(2) All dietary scenarios have a standardized energy content for both men (2718 kcal/day/capita) and women (2033 kcal/day/capita) that are based on the published data of the HDNSS (Supplement 4). Separation of male and female scenarios was necessary, since the recommended nutrient values are gender specific ones, and the published data of the HDNSS—which were the bases of all scenarios—are also specific for genders [33].
(3) The food group of alcoholic drinks was included in all scenarios even if they were not included in all of the alternative guidelines, since they are present in the nutrition of the Hungarian population in a considerable amount [31] and the exclusion of them from alternative dietary scenarios would show biased results.

### 2.3. Water Footprint of the Food Groups

As an environmental impact factor, the water footprint was selected for the present study. As it was mentioned, food production is responsible for the majority of anthropogenic water use [5]. To achieve smaller water footprint of food products is a globally important issue and the concept of water footprint helps to make more environmentally conscientious decisions to this direction. The water footprint includes three main components: Blue, green, and gray water footprints. The gray water footprint was excluded from the calculations because national data was partly lacking and because it is rather a qualitative indictor of water use. The blue water footprint represents water sourced from surfaces or groundwater for irrigation, and industrial and domestic use. Green water footprint is water from precipitation that is stored in the root zone of the soil and evaporated, transpired, and incorporated into a product. In particular, it is the water type relevant for agricultural, horticultural, and forestry production [42]. The data on the water footprint were acquired from the Water Footprint Network (WFN) for both plant-based [43] and animal-based foods [44]. The definition and dataset of the WFN on the water footprint are widely applied in similar studies on sustainable nutrition [18,19,20,21,22,23,24,30,45]. The WFN was also preferred since it contains data on national and subnational levels that can make the results more specific for a country. Recent studies have calculated and recommended the inclusion of the green water footprint since it represents the greatest water consumption measured in quantity [21,22,23,30]. Studies on sustainable nutrition have either included all types of water footprints in the calculation [20,22,30,45] or analyzed them separately [30].

**Figure 3:**
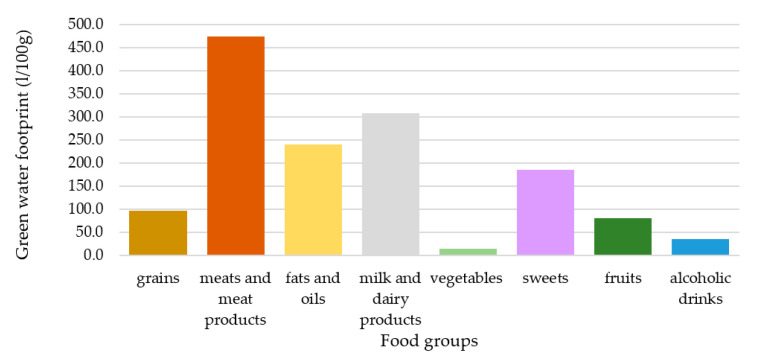
Weighted average green water footprint of food groups.

**Figure 4:**
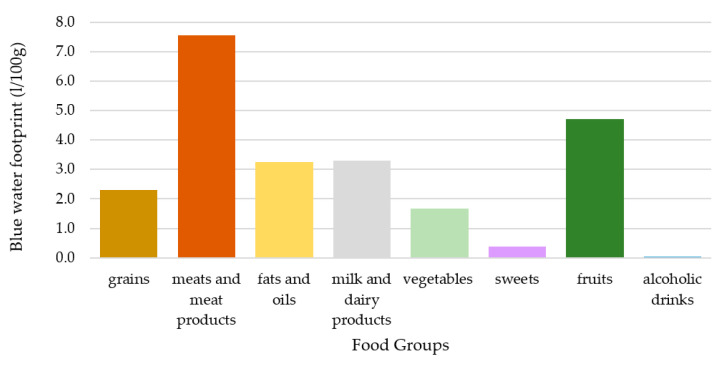
Weighted average blue water footprint of food groups.

In this study, both green and blue water footprints were analyzed separately. The inclusion of the green water footprint was suggested by Vanham [30] and it is the most relevant in terms of volume. The blue water footprint was included in all reviewed studies and it was analyzed separately in this present study, since it can lead to controversial results because the relative ranking of food groups differs in the case of blue and green water footprints, as can be seen in Figure 3 and Figure 4. This is mainly due to the relatively large blue water footprint of fruits [24]. Fish is a problematic element in the case of the water footprint assessment and is sometimes left out due to a lack of data [23]. However, in this study fish was given a value of 0 for its green water footprint, based on its definition, and valued according to published data regarding its blue water footprint [24]. In this present analysis, the unit of the water footprint of food groups (m^3^/tonne/food item) was transformed to a L/100 g/food group dimension in the scenarios, since all the other variables referred to a 100 g/food group unit. The weighted average of the blue and green water footprints (*FW_p_*) of the different food groups were calculated in the same way as in the case of the weighted average nutrient density values: The amount of national supplies was considered as the weight factor (Supplement 1).

(2) $$FW_{p}=\frac{\sum_{i=1}^{i=n}FI_{Si}WD_{i}}{\sum_{i=1}^{i=n}FI_{Si}}$$

where:

*FG_wf_* = weighted average of water footprint of the pth food group (L/100 g/day/cap),
*FI_s_* = supply of food item (100 g/day/cap), and
*WD_i_* = water footprint of food item [L/100 g].

### 2.4. Integrated Analysis of Scenarios

**Figure 5:**
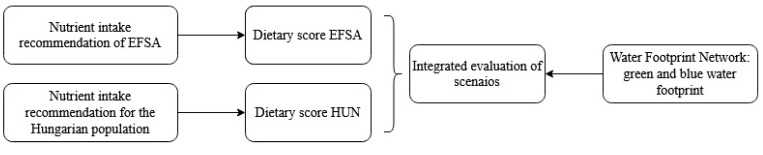
Flowchart of the integrated analysis of scenarios. (EFSA: European Food Safety Authority, HUN: Hungary).

According to the principle of this present study, the scenarios were analyzed from an integrated perspective of sustainable nutrition. This included healthiness (i.e., dietary quality) and sustainability (i.e., blue and green water footprint) and whether there are synergies between them (Figure 5).

### 2.5. Development and Application of Dietary Quality Score

Dietary quality scores have become a useful tool for the integrated analysis of sustainable nutrition, which includes both health and environmental aspects. Dietary quality scores can refer to foods, meals, and diets and give a measure of dietary quality (i.e., healthiness) [10]. However, there are different approaches to calculate dietary quality scores, so the method depends on the overall aim of the given study. One approach is to only consider “health indicators” (focusing on a relatively low number of nutritional components that usually indicate the good quality of a diet) in the scores [46,47]; another approach is to include a large number of nutrients [48,49,50,51]. Gazan et al. [29] concluded that including just a few nutrients can lead to misleading results. Dietary quality scores are mostly based on algorithms related to nutrient density values. Algorithms are most often calculated using the ratio of the dietary reference value and the actual nutrient density. Another approach is to evaluate the dietary quality on the basis of the fulfilment of different criteria [10]. In the present study, both types of calculations were included in the algorithms. To ensure the suitability of the dietary quality score, two different scores were developed:

(1) Dietary quality score_HUN_: The algorithms of this score were based on the dietary reference values for the healthy Hungarian population published in Sarkadi Nagy et al. [33] (Supplement 5).
(2) Dietary quality score_EFSA_: The algorithms of this score were based on the dietary reference values for the healthy population published by EFSA [52,53] (Supplement 5).

Both dietary quality scores are a sum of scores referring to each single nutrient. In order to differentiate nutrients based on their type of recommendation and their role in the population’s health, we created four groups (Table 3). The classification of dietary quality scores into subscores can ensure its suitability as a tool to describe healthiness, as it has also been applied in similar studies [12,54]. The main reason to classify subgroups is that nutrient density values simply cannot differentiate which nutrient intake level is relatively “good” or “bad” at a population level. The algorithms and classifications fundamentally determine the applicability of a dietary quality score so its design should be based on a comprehensive literature review. The detailed description of the subscores can be found in Supplement 3 and Supplement 5. In the interpretation of the results of an integrated dietary quality value (IDQV) was applied. This was calculated on basis of the dietary quality score_EFSA_ and the dietary quality score_HUN_ (Supplement 3). In the case of both scores, the baseline scenario was considered the reference point and all other scenarios were measured according to their deviation in % from this point. The final value is the average deviation of the scenarios in % from the HDNSS original of the dietary quality score_EFSA_ and the dietary quality score_HUN_. According to this calculation, the value of the integrated dietary quality value of the baseline scenario is 0. Those scenarios characterized by a “−“value are worse, and those by a “+” are better than the HNDSS-original scenario (Supplement 3).

## 3. Results

### 3.1. The Relationship between Water Footprint and Dietary Quality

Dietary scenarios were analyzed along two dimensions: Water footprint and dietary quality. The water footprint is measured in L/capita/day and dietary quality is represented by the integrated value of the dietary quality score_HUN_ and the dietary quality score_EFSA_ (IDQV) (or the detailed scores, look for S6). There are four different analyses classified by gender and type of water footprint: (1) Blue water footprint (GWF) in female scenarios, (2) blue water footprint (BWF) in male scenarios, (3) green water footprint in female scenarios, and (4) green water footprint in male scenarios.

**Figure 6:**
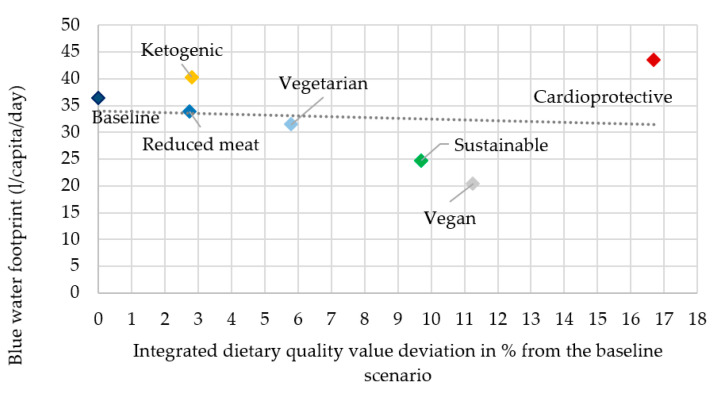
Blue water footprint in female scenarios.

**Figure 7:**
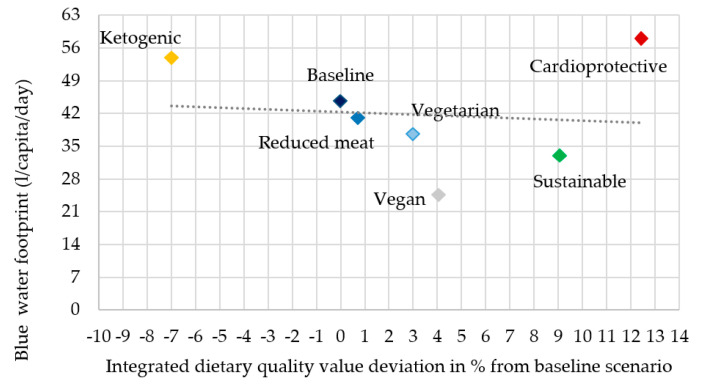
Blue water footprint in male scenarios.

**Figure 8:**
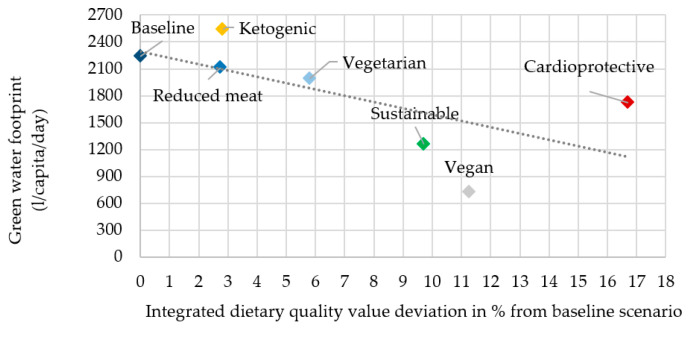
Green water footprint in female scenarios.

**Figure 9:**
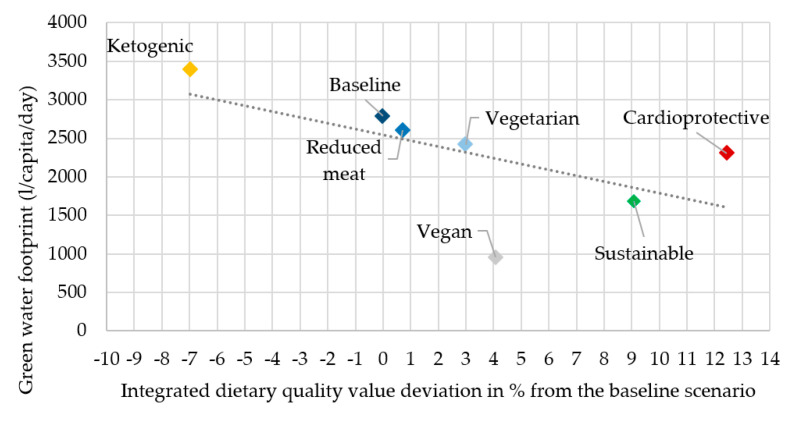
Green water footprint in male scenarios.

In the description, the rank of the scenarios refers to the most advantageous as first and the most disadvantageous as seventh in both dietary quality and water footprint (Figure 6, Figure 7, Figure 8 and Figure 9).

Based on the integrative approach, regarding both the water footprint and dietary quality, the vegan (second in IDQV: +11.3% and first in BWF: 20.4 L/capita/day) and the sustainable (third in IDQV: +9.7% and second in BWF: 24.7 L/capita/day) scenarios were the most advantageous. The high vegetable and grain and no animal-based food content of the vegan scenario and the high vegetable, grain, and fruit content, and the moderate milk and dairy product, and meat, fat, and oil content of the sustainable scenario can explain these results. The cardioprotective scenario was first for dietary quality value (+16.7%) but seventh for its blue water footprint (43.4 L/capita/day), due to its high fruit content, which contributes significantly to the blue water footprint. The high fruit content also contributes to a high IDQV, as it is typically high in qualifying nutrients and low in disqualifying nutrients. The baseline scenario representing the current Hungarian nutrition was seventh in dietary quality value and fifth in blue water footprint (36.3 L/capita/day). Compared to the baseline scenario, the reduced meat and vegetarian scenarios were lower in blue water footprint (fourth and third with 33.9 and 31.5 L/capita/day) and higher in dietary quality value (sixth with +2.7% and fourth with +5.8%) but not by as much as expected, probably because only the meat group was modified and the scenarios were still low in vegetables and fruits. The ketogenic scenario was disadvantageous, being sixth in terms of its water footprint (40.2 L/capita/day) and fifth in dietary quality (+2.8%). This result of the ketogenic scenario was clearly disadvantageous due to its high fat, oil, and meat content and low fruit and grain content (Figure 6).

Similar to the female scenarios, the cardioprotective scenario was the seventh in its blue water footprint (58 L/capita/day) and first in dietary quality value (+12.4%). These results occur for the same reason as in the case of female scenarios: A high fruit content with relatively high grain and vegetable content. The sustainable scenario was the second highest in IDQV (+9.1%) and second lowest in blue water footprint (33 L/capita/day); thus, it was the most favorable scenario in this analysis. This scenario is high in fruits, vegetables, and grains, while moderate in meats, milk and dairy products, and fats and oils. The vegan scenario showed less favorable results than it did in the female scenarios, ranking third in IDQV (+4.1%); however, it was still first lowest in blue water footprint (24.5 L/capita/day). Compared to female scenarios, male scenarios are denser in energy and nutrients, which can result in different rankings in IDQV. Baseline and reduced meat scenarios showed very similar results in both aspects (IDQV: sixth with 0% and fifth with +0.7%, BWF: fifth with 44.6 and fourth with 41 L/capita/day), probably because in the reduced meat scenario meats were partly replaced with animal based-food that has similar characteristics in nutrient density and also has a high blue water footprint. The vegetarian scenario ranked fourth in IDQV (+3%) and third in blue water footprint (37.5 L/capita/day). The reason that this scenario is not more advantageous is that only the meats’ group was modified and replaced by animal-based foods, nuts, and legumes, and it was still low in other vegetables and fruits. The ketogenic scenario was the most disadvantageous in both dimensions: seventh in IDQV: −7% and seventh BWF: 53.8 L/capita/day (Figure 7).

As was described in the introduction, blue and green water footprint in scenarios may show controversial results and this was also proven in this present analysis. Besides its high dietary quality value (first in IDQV: +16.7%), the cardioprotective scenario’s green water footprint (GWF: third with 1724.4 L/capita/day) is also advantageous. The vegan (second in IDQV: +11.3%, first in GWF: 729.8 L/capita/day) and sustainable (third in IDQV: +9.7%, second in GWF: 1257.9 L/capita/day) scenarios also showed promising results in this analysis. There were considerable differences between the original (seventh in IDQV: 0%, sixth in GWF: 2238.7 L/capita/day), reduced meat (sixth in IDQV: +2.7%, fifth in GWF: 2114 L/capita/day), and vegetarian (fourth in IDQV: +5.7%, fourth in GWF: 1989.2 L/capita/day) scenarios in IDQV; however, there was only a slight difference in GWF. Baseline and ketogenic (fifth in IDQV: +2.8%, seventh in GWF: 2538 L/capita/day) scenarios were the most disadvantageous scenarios overall, ranked as worsts in both aspects. In the case of green water, the animal-based food content clearly determined the rank of scenarios in terms of their overall water footprint. Foods with a relatively high animal-based content also make a great contribution to a low IDQV since they are high in disqualifying nutrients such as saturated fatty acids, cholesterol, sodium, and total lipids (Figure 8).

Compared to the blue water footprint, the green water footprint of the cardioprotective scenario was more advantageous (third in GWF: 2305.4 L/capita/day, first in IDQV: +12.4%), which can be explained by the same reasons as with the female scenarios. The most advantageous scenario was the sustainable one, ranked second for both its green water footprint (1681.7 L/capita/day) and its dietary quality (IDQV: +9.1%); this can also be explained by the same reasons as the female scenarios. The vegan scenario was third in dietary quality (IDQV: +4.1%) and first in green water footprint (954.7 L/capita/day). The baseline (sixth in IDQV: 0% and sixth in GWF: 2785.6 L/capita/day), reduced meat (fifth in IDQV: +0.7% and fifth in GWF: 2602.1 L/capita/day), and vegetarian (fourth in IDQV: +3% and fourth in GWF: 2418.5 L/capita/day) scenarios were similar to the blue water footprint analyses of male scenarios because they were not considerably different, either in their green water footprint or in dietary quality. In this assessment, similar to the blue water footprint, the male, ketogenic scenario was described as most disadvantageous in both aspects (seventh in IDQV: −7% and seventh in GWF: 3393.2 L/capita/day) (Figure 9).

Authors have tested stochastic relationships between the dietary quality values and types of water footprint in the different scenarios. For this purpose, linear regression models were fitted. The only significant relation could be proven for green water footprint in male scenarios (Figure 9.). In this case, a significant, negative relationship between dietary quality values and green water footprints could be proven, but the coefficient of correlation is rather low (*r*^2^ = 0.7; *p* = 0.2).

### 3.2. Analysis of the Relative Changes in the Water Footprint in Different Scenarios

In this analysis, the reference scenario was also the baseline scenario since it represents the current Hungarian nutrition. In the case of the green water footprint, in both female (+13.4%) and male (+21.8%) scenarios only the ketogenic scenarios deviated positively. All other scenarios resulted in a greater or lesser reduction in the green water footprint. In the case of the blue water footprint, beside ketogenic scenarios (female +10.9%, male +20.7%), cardioprotective scenarios (female +19.6%, male +30.1%) also resulted in an increase, for both female and male scenarios. However, regarding the green water footprint, cardioprotective scenarios also resulted in a considerable decrease (female −23%, male −17.2%). All other scenarios resulted in a greater or lesser decrease in the blue water footprint. Vegan scenarios resulted in the greatest decrease in both green (female −67.4%, male −65.7%) and blue water footprints (female −43.8%, male −45%). Following vegan scenarios, the sustainable scenario also resulted in a considerable decrease in both green (female −43.8%, male −39.6%) and blue water footprints (female −31.9%, male −26%). The reduced meat (female: BWF: −6.6%, GWF: −5.6%, male BWF: −8%, GWF: −6.6%) and vegetarian (female: BWF: −13.3%, GWF: −11.1%, male BWF: −15.9%, GWF: −13.2%) scenarios also reduced both green and blue water footprints and this decrease was mainly determined by the reduction of the meat content (Table 4).

## 4. Discussion

### 4.1. Synergy between Dietary Quality and Environmental Impact

The healthiness and environmental burden of a diet are two different dimensions, which is why, as a general rule, we cannot deduce one from the other, with the exception of meat products: In the case of this product group, due to technology, the water demand is much higher than for other food products. As stated, a clear stochastic relationship cannot be proven between sustainability and health, but a reduction in the intake of animal-based foods would generally decrease the environmental burden of nutrition [7,11,13,31]. One main goal of the present study was to analyze synergies between the healthiness and sustainability of nutrition in the context of the typical Hungarian nutrition. Several similar studies have analyzed this synergy, focusing on different populations, using different metrics for environmental impact [10,11,13,57]. It is still not clear whether there is a definite association between dietary quality and environmental impact [29]. Given that this issue is enormously complex, with numerous contributory factors, the results are somewhat dependent on the sophisticated details. As already mentioned, the different environmental factors are in correlation, so rough comparisons can be based on the results of other environmental impact categories. The most frequently applied factor is GHGE, which serves as an indicator for environmental impact [47]. According to the review on sustainable nutrition by Hallström et al. [11], the reduction in GHGE in vegetarian scenarios compared to current nutrition is about 20–35%, and in vegan scenarios 25–55%. In this present study, considering both female and male and green and blue water footprints, the reduction of the water footprint in vegetarian scenarios was between 11.1–15.9%, and in vegan scenarios between 43.8–67.4%. The variation in the results is due to the different environmental impact categories and different methodology used to create and evaluate scenarios; however, there is no question that the fewer animal-based food features in the scenarios, the more sustainable they are.

In this study, the most advantageous scenario was the sustainable one, based on the Planetary Healthy Diet published by Willett et al. [39]. This scenario contains a large amount of grains, vegetables, and fruits and a moderate amount of meats, milk and dairy products, fats and oils, alcoholic drinks, and sweets. Vegan, vegetarian, and reduced meat scenarios were more advantageous than the scenario that represented the current Hungarian nutrition (baseline scenario). Other studies that analyzed the sustainable nutrition of other populations in Europe have drawn similar conclusions [22,23,24,30,58]. However, the situation is not as simple as claiming that the smaller the water footprint the healthier the nutrition, since any more detailed analyses of the water footprint show a more controversial picture [18,19,22,24]. This study also supported these facts and the separate analysis of green and blue water showed a somewhat controversial picture. The cardioprotective scenario also had the most synergetic characteristics: Its green water footprint was lower and it was healthier than the current nutrition (baseline); however, in the case of the blue water footprint, the opposite was true. The advantages of the cardioprotective diet in terms of sustainability have already been supported by [25]. The ketogenic (i.e., a low-carbohydrate, high-fat) diet is one of the most popular alternative diets nowadays; however, its high ecological impact is rarely analyzed in the way it has been by Röös et al. [37]. This present study also proved that the ketogenic diet is not a means of ensuring sustainable nutrition for the future. They concluded that the ketogenic diet has a higher environmental impact (climate impact, loss of biodiversity, land use) than current Swedish nutrition (+28%) and the Nordic recommended nutrition. In this present study, the increase in the water footprint was also considerable in the ketogenic scenarios (female: GWF: +13.4%, BWF: +10.9% and male: GWF: +21.8% and BWF: +20.7%), although the assessed environmental impact category was different.

### 4.2. Reduction of the Water Footprint: Comparison of Results with Other Studies 

When drawing conclusions on the reduction in the water footprint in the different scenarios, we separated green and blue water footprints, as they were analyzed separately in the present study. The results of the green water footprint will be compared to studies that have analyzed either the green water footprint separately or the total water use that involves both types of water. In terms of volume, total water use is similar to the green water footprint since it represents the largest proportion [21,22]. De Marco et al. [20] calculated a negative association between a Mediterranean diet adequacy index and the water footprint, and Capone et al. [21] calculated a 69.9% reduction in the total water footprint in the case of a shift to a Mediterranean diet from the current Italian diet. In this study, the sustainable and cardioprotective scenarios most resembled the Mediterranean diet and they also resulted in a considerable decrease in the green water footprint (female scenarios: −39.6% and −23.0%, male scenarios: −43.8% and −17.2%) compared to the current Hungarian nutrition. In the case of reduced meat and plant-based diets, Vanham et al. [22] calculated a 27% reduction in the total water footprint in the Eastern-Central European region, including Hungary, while in this study the reduction in the GWF was −5.6–67.4% in the female- and −13.2–65.7% in the male-related scenarios. In the case of the green water footprint and total water use, the amount of animal-based food had the greatest effect on the results.

Considering the results of the analysis of the blue water footprint and healthiness, the picture is more controversial and the stochastic relationship cannot exactly be proven; however, in terms of volume, the use of blue water is lower compared to green water. As has been described by Tom et al. [19], reducing meat intake could lower the environmental impact of nutrition; however, if we replace it with other high environmental impact food groups, this effect can vanish. They carried out an analysis of the population of the United States and found that if they shifted from their current to the recommended nutrition, the blue water footprint would decrease by 10%. In the present study, the shift from current nutrition to sustainable nutrition would also result in a decrease in the blue water footprint (female scenario: −31.9%, male: −26%). However, proving the argument made by Tom et al. [19] in the case of cardioprotective scenarios, a partial replacement of meat with a high amount of fruit resulted in an elevated blue water footprint (female: +19.6%, male +30.1%). Hess et al. [18] concluded that the shift in nutrition based on vegetarian and healthy scenarios in the UK population would only result in an insignificant change in the blue water footprint (−4–8%). In summary, regarding the blue water footprint, the picture is not as simple as to suggest that a reduction in animal-based food would directly lead to a lower blue water footprint, but a more complex change in nutrition could save blue water, as was proven in this study in the case of the sustainable scenario.

### 4.3. Differences between the Genders

The differences between the two genders in the analyses are mainly based on the fact that there were considerably different scenarios for them. Male scenarios were standardized to 2718 kcal while female scenarios to 2033 kcal, according to the published data of the HDNSS. The different rankings of scenarios in their health scores derived from the fact that the nutritional reference values were different for the two genders. In the case of scenarios where extreme upper and lower nutrient values were calculated (ketogenic and vegan), the results were proportionally more different due to the considerable impact of the initial energy density values. Also, there were greater differences in the water footprint of scenarios in the male, compared to the female, scenarios. Again, this derived from the simple fact that the energy density had a great effect on the size of the water footprint, since the more we eat, the more water is used for food production. In summary, regardless of the detailed analysis of green and blue water footprints and genders, sustainable scenarios were the most advantageous. Meier and Christen [24] analyzed the difference between the genders, although they applied a quite different approach. They concluded that the blue water use of food consumption was very similar for both genders, considering that, in the case of other environmental impact factors (i.e., GHGE, land use, NH_3_ emission), this difference was greater between the two genders. The reason for this lies in the structure of food consumption; while men consume more animal-based groups, women tend to consume more fruits, whose contribution to blue water use is considerable.

### 4.4. Limitations

The limitations that define the conclusions we can draw are the following.

(1) We used secondary data for our calculation; however, the data on the water footprint, food consumption (Central Statistical Office (CSO) [32]), food supply (Food and Agriculture Organization (FAO) [31]), and the nutritional survey by Sarkadi Nagy et al. [33] were all country specific. The only noncountry-specific data we used were acquired from the FoodData Central Database of the USDA [36], but this is a widely accepted source in nutrition science. Similar studies that analyzed sustainable nutrition on a population have also mostly used secondary data [10,11,57].
(2) The scenarios are “theoretical” diets that are based on the current nutrition pattern of Hungary. In studies that analyze dietary scenarios, there is always a question as to how realistic they are. “Cultural acceptability” is a very important aspect of sustainable nutrition [9] and even though dietary quality and sustainability are crucially important for the future, we cannot map out a pathway for future nutrition that is not regionally acceptable. In this study, we ensured cultural acceptability with the food items included, all of which were the most commonly consumed food items weighted by their supply value according to our national statistical data [32].
(3) Another limiting aspect of this present and other similar studies is comparability. However, these comparisons have been made with the limitations based on the following differences: (1) The type of environmental impact categories included in the calculations, (2) the creation and evaluation of the dietary scenarios, and (3) the population analyzed.
(4) The availability of data in some cases has been rather limited. It would be highly desirable to use more recent data, but the current international and national statistical systems are not capable of offering more recent information.

## 5. Conclusions

A diet based on the principle of moderation could be a future direction for the nutrition of the Hungarian population to follow. In the present study, the overall, most advantageous scenario was the sustainable scenario, which was based on the planetary health diet published by Willett et al. [39] and was adapted to the Hungarian population with the inclusion of the most commonly consumed foods in Hungary. This is a balanced dietary scenario, based on a moderate amount of meat, milk and dairy products, fats and oils, sweets, and alcoholic drinks, while containing a high amount of grains, vegetables, and fruits. The cardioprotective diet proved to be a healthier (as it is the preventive diet of the leading cause of mortality in Hungary (Institute for Health Metrics and Evaluation (IHME) [38]) and a more sustainable scenario compared to recent nutrition; however, there are aspects that must be considered regarding its blue water footprint and optimization. Even though vegan scenarios had the lowest water footprint, they cannot be recommended on a population level due to their nutritional inadequacy; they would also not be in any way culturally acceptable since they would represent a sharp shift away from recent nutrition patterns. A vegetarian diet could be adequate for most of the healthy and adult population with a lower water footprint, but would still result in a great shift in nutrition. This study has also shown that merely exchanging meat for other animal- and plant-based foods high in protein in vegetarian scenarios did not necessarily result in high dietary quality, so other changes would also be necessary, such as a higher vegetable intake. Because of this, people who willingly adhere to a vegetarian or vegan diet should be able to get every possible professional assistance to keep a suitable diet, as recommended by the FAO [5].

As was the aim of this study, it gives supporting evidence for the future on how to create dietary guidelines for the Hungarian population, which include sustainability. Our results could support nutrition-related education and personalized nutrition, which is extremely important in providing nutrition care [59]. The detailed analysis of the water footprint showed that adaptation to the planetary healthy diet could be the most advantageous among the analyzed scenarios since it has the best synergistically characteristic features regarding both health and sustainability. Thus, Hungary could follow the footsteps of those countries that have included sustainability in their dietary guidelines [5,8,26,27]. The further investigations in the field should be devoted to development of research of links between diet nutrition and quality of life, particularly poverty, and overall well-being as one of the key features of sustainable development, defined in [60,61,62].

## Figures and Tables

**Table 1:** Some relevant studies regarding the association between dietary quality and water footprint.

Study, Year, Country	Ecological Indicator	Dietary Quality Assessment	Nutritional Data	Key Findings
Hess, et al. [18], UK	blue water footprint	food consumption quantity and structure as an indicator of dietary quality	five alternative healthier diet scenarios based on UK food consumption	Healthier scenarios had a small impact on total blue water scarcity footprint.
Tom, et al. [19], US	energy use, blue water footprint, and greenhouse gas emissions	foods as an indicator of dietary quality	three dietary scenarios based, on the 2010 USDA Dietary Guidelines	Reducing caloric intake levels to achieve “normal” weight without shifting food mix decreases energy use, blue water footprint and GHG emissions by around 9%.
De Marco, et al. [20], European perspective	ecological, water and carbon footprints	food consumption quantity and structure as an indicator of dietary quality, Mediterranean Adequacy Index (MAI)	food consumption data from the Food and Agriculture Organization food balance sheets in 48 European countries	An increase of 1 unit of MAI can reduce the ecological, carbon and water footprint
Capone, et al. [21], Italy	water footprint	food consumption quantity and structure as an indicator of dietary quality	food consumption data from the Food and Agriculture Organization food balance sheets in Italy, the United States and Finland	Adherence of the Italian population to the Mediterranean dietary pattern can bring health benefits and also reduces water footprint.
Vanham, et al. [22], Austria	water footprint	food consumption quantity and structure as an indicator of dietary quality	four different scenarios based upon food-based dietary guidelines	A vegetarian diet would result in the lowest water footprint consumption.
Vanham, et al. [23] European perspective	water footprint	food consumption quantity and structure as an indicator of dietary quality	four different scenarios based upon regional food-based dietary guidelines	A vegetarian diet could result in lower water use related to food consumption in European countries.
Meier and Christen [24]	chemical emissions, land use, blue water use	food consumption quantity and structure as an indicator of dietary quality	separated analysis of different food groups	In the case of sustainable food consumption gender is an important factor to consider.
Downs and Fanzo [25]	carbon footprint, ecological footprint, water footprint	food consumption quantity and structure as an indicator of dietary quality	separated analysis of different food groups	A cardioprotective diet can contribute to a more healthy and sustainable diet; however in order to achieve this food choices need to be considered specifically.

**Table 2:** Characteristics of the different dietary scenarios.

Scenarios	Short Description	Data Source
(1) Dietary Scenarios Based on the HDNSS and its Modifications		
Baseline (HDNSSoriginal)	The baseline scenario represents the current nutrition in Hungary; the proportions of food groups (kcal/capita/day) are based on the published data of the HDNSS.	Sarkadi Nagy, et al. [33]
Reduced meat content diet	The reduced meat scenario is based on the baseline scenario; the meat food group was reduced by 50% in kcal and was replaced by eggs (12.5% in kcal), dairy products (12.5% in kcal), legumes (12.5% in kcal) and nuts (12.5% in kcal).	Sarkadi Nagy, et al. [33]
Vegetarian diet	The vegetarian scenario is based on the baseline scenario; the meat food group was reduced by 100% and was replaced by eggs (25% in kcal), dairy products (25% in kcal), legumes (25% in kcal) and nuts (25% in kcal).	Sarkadi Nagy, et al. [33]
Vegan diet	The vegan scenario is based on the baseline scenario; the meat and milk and dairy products food groups were reduced by 100% and replaced by grains (25% in kcal), potatoes (25% in kcal), legumes (25% in kcal) and nuts (25% in kcal).	Sarkadi Nagy, et al. [33]
**(2) Dietary Scenarios Based on Guidelines and Alternative Diets**		
Planetary health diet (Sustainable)	The sustainable scenario is based on the description of the planetary health diet. The planetary health diet is developed on the principle of respect for health and nature.	Willett, et al. [39]
Cardioprotective diet (Cardioprotective)	The cardioprotective scenario is based on the elements of the cardioprotective diet.	Mozaffarian, et al. [40]
Low-carbohydrate high-fat diet (Ketogenic)	The ketogenic scenario is based on the widely accepted nutrient distribution of low-carbohydrate high-fat diets: 50–60% fat, 20–30% protein and a maximum of 30% carbohydrates	Adam-Perrot, et al. [41]

**Table 3:** The classification of nutrients and dietary quality scores.

Classification of Nutrients	Elements of Dietary Quality Score_HUN_ (*n* = 25)	Elements of Dietary Quality Score_EFSA_ (*n* = 20)
(1) Qualifying nutrients (The population intake level of these is either adequate or low and a reasonably higher intake level is not related to health-risks). To elevate their intake would be beneficial on the population level) [34,52,53,55].	dietary fiber (g), thiamin (mg), riboflavin (mg), niacin (NE), vitamin B6 (mg), folate (μg), vitamin B12 (μg), vitamin C (mg), vitamin A (μg), vitamin E (mg), calcium (mg), magnesium (mg), zinc (mg), potassium (mg), iron (mg), phosphorus (mg)	dietary fiber (g), thiamin (mg), riboflavin (mg), niacin (NE), vitamin B6 (mg), folate (μg), vitamin C (mg), vitamin A (μg), calcium (mg), magnesium (mg), zinc (mg), potassium (mg), iron (mg), phosphorus (mg)
(2) Dis-qualifying nutrients (the population intake level of these is high and related to health-risks) [34,35,52,53,55]. To lower their intake would be beneficial on the population level.	sugars (g), cholesterol (mg), total fat (g), sodium (mg), saturated fatty acids (mg)	sugars (g), saturated fatty acids (mg)
(3) Macronutrients with recommended intake range (nutrients that contribute to energy intake and have a recommended reference range) [56].	total carbohydrate (g), total protein (g)	total carbohydrate (g), total protein (g), total fat (g)
(4) Recommended intake ratio of two nutrients (nutrients that interfere with each other in their absorption and/or utilization [34,35,55].	Na:K, Ca:P	

**Table 4:** Comparison of different scenarios on the basis of their green and blue water footprints.

Scenarios	Green Water Footprint		Blue Water Footprint	
Male	Value (L/Capita/Day)	Change in % Compared to Baseline Scenario	Value (L/Capita/Day)	Change in % Compared to Baseline Scenario
**Baseline**	2785.6		44.6	
**Reduced meat**	2602.1	−6.6	41	−8
**Vegetarian**	2418.5	−13.2	37.5	−15.9
**Vegan**	954.7	−65.7	24.5	−45
**Sustainable**	1681.7	−39.6	33	−26
**Cardioprotective**	2305.4	−17.2	58	+30.1
**Ketogenic**	3393.2	+21.8	53.8	+20.7
**Female**				
**Baseline**	2238.7		36.3	
**Reduced meat**	2114	−5.6	33.9	−6.6
**Vegetarian**	1989.2	−11.1	31.5	−13.3
**Vegan**	729.8	−67.4	20.4	−43.8
**Sustainable**	1257.9	−43.8	24.7	−31.9
**Cardioprotective**	1724.4	−23	43.4	+19.6
**Ketogenic**	2538	+13.4	40.2	+10.9

## Supplementary Materials

The following are available online at https://www.mdpi.com/2072-6643/12/9/2578/s1, Supplement 1: Detailed list and weights of food items, Supplement 2: Proportion of the different food groups in the scenarios, Supplement 3: Detailed description of qualifying and dis-qualifying nutrients, Supplement 4: Detailed description of the reference humans, Supplement 5: Details of the recommended dietary intake values,

## Abbreviations

GHGE	greenhouse gas emission
GWF	green water footprint
BWF	blue water footprint
IDQV	integrated dietary quality value
FAO FBS	Food and Agriculture Organization of the United Nations Food Balance Sheet
HDNSS	Hungarian Diet and Nutritional Status Survey
CSO	Central Statistical Office
IHME	Institute for Health Metrics and Evaluation
WFN	Water Footprint Network
