# Supplementary material for: Is the Sustainable Choice a Healthy Choice?—Water Footprint Consequence of Changing Dietary Patterns
Source: Nutrients. 2020 Aug 25;12(9):2578. doi: 10.3390/nu12092578 (PMC7551173; doi:10.3390/nu12092578)
Supplement: Supplementary file 1 [file nutrients-12-02578-s001.pdf]

Supplement 1

Food groups	Food items	Supply (g/day/capita)
**Based on the classification of HDNSS, 2014**	The supply of the food items is mostly based on the FAO FBS and specified with the database of the Central Statistical Office of Hungary. In the calculation of the average water footprint and nutrient values of the scenarios, supply quantities were used as weight in a 1:1 ratio. In the case of "Fruits, others and Vegetables, others" the simple average was calculated based on the most commonly consumed food items.	
**Grains**	Wheat and products	301
	Rice (Milled Equivalent)	6
	Rye and products	4
**Meats and meat products** (including eggs)	Pig meat	96
	Poultry Meat	65
	Eggs	34
	Bovine Meat	14
	Freshwater Fish	7
	Offals, Edible	4
	Fish	14
**Fats and oils**	Fats, Animals, Raw	35
	Sunflower Seed Oil	30
	Palm Oil	13
	Rape and Mustard Oil	6
	Soybean Oil	4
**Milk and dairy products**	Milk - excluding Butter (-yoghurt and cheese)	388
	Cheese	16
	Yoghurt	32
	Cream	18
**Vegetables**	Vegetables, Other	158
	Potatoes and products	127
	Tomatoes and products	41
	Onions	18
	Peas	6
	Vegetables, other:	
	Leafy vegetables	
	Cabbage	
	Cucumber	
	Green pepper	
	Beans	
	Carrot	
**Sweets**	Sugar (raw equivalent)	52

	Sweeteners, Other	43
	Cocoa Beans and products	7
**Fruits**	Fruits, Other	64
	Apples and products	29
	Oranges, Mandarins	29
	Grapes and products (excl. wine)	16
	Bananas	9
	Citrus, Other	4
	Pimento	4
	Nuts and products	4
	Fruit, others:	
	Apricot	
	Peach	
	Cherry/sour cherry	
	Berries	
	Plum	
	Pear	
	Watermelon	
	Nuts	
	Raisin	
**Alcoholic drinks**	Beer	176
	Wine	66
	Spirits	17

Sources: [1], [2], [3]

References:

1. Sarkadi Nagy, E.; Bakacs, M.; Illés, É.; Nagy, B.; Varga, A.; Kis, O.; Schreiberné Molnár, E.; Martos, É., Országos Táplálkozás és Tápláltsági Állapot Vizsgálat–OTÁP2014. II. A magyar lakosság energia-és makrotápanyag-bevitele. *Orv. Hetil.* **2017,** 158, (15), 587-597. https://doi.org/10.1556/650.2017.30718
2. Central Statistical Office (CSO) *2.2.3.6. Amount of food consumption per capita per year classified by income and type of region into deciles (2010-);* 2013. https://www.ksh.hu/docs/hun/xstadat/xstadateves/i_zhc023a.html (Accessed on: 20 June 2020)
3. Food and Agriculture Organization (FAO) *Food Balance Sheet;* 2013. http://www.fao.org/faostat/en/#data/FBS (Accessed on: 20 June 2020)

Supplement 2

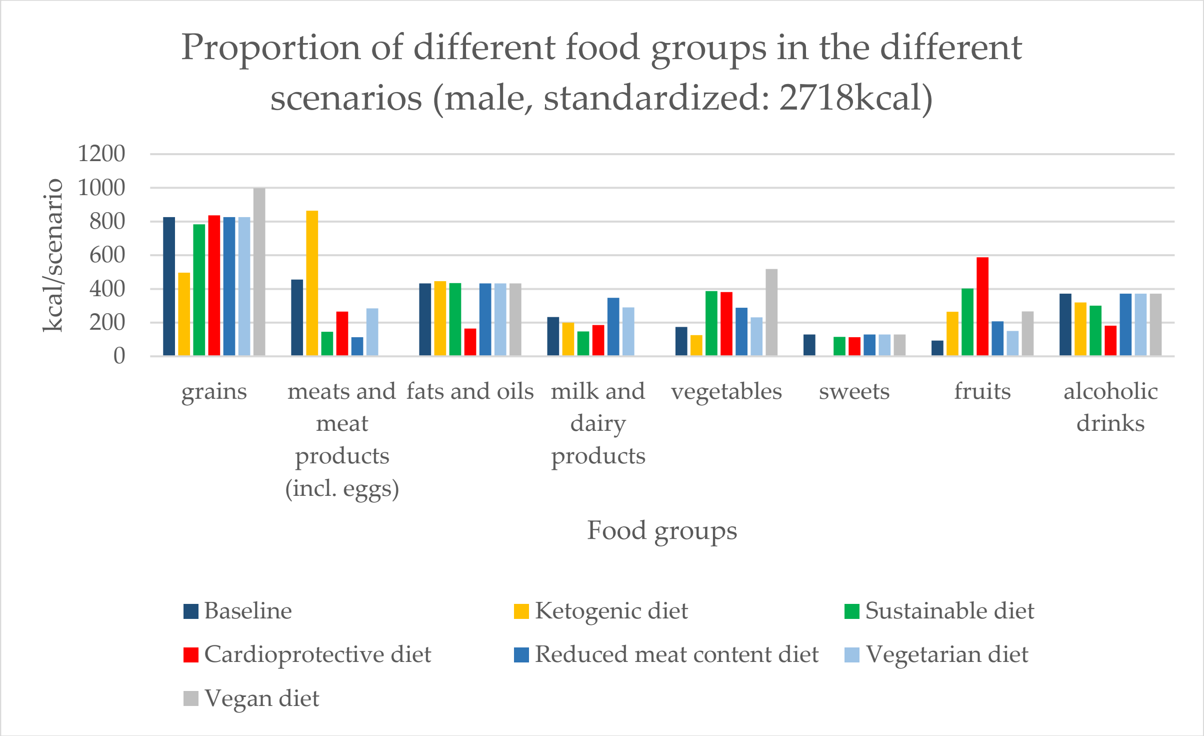

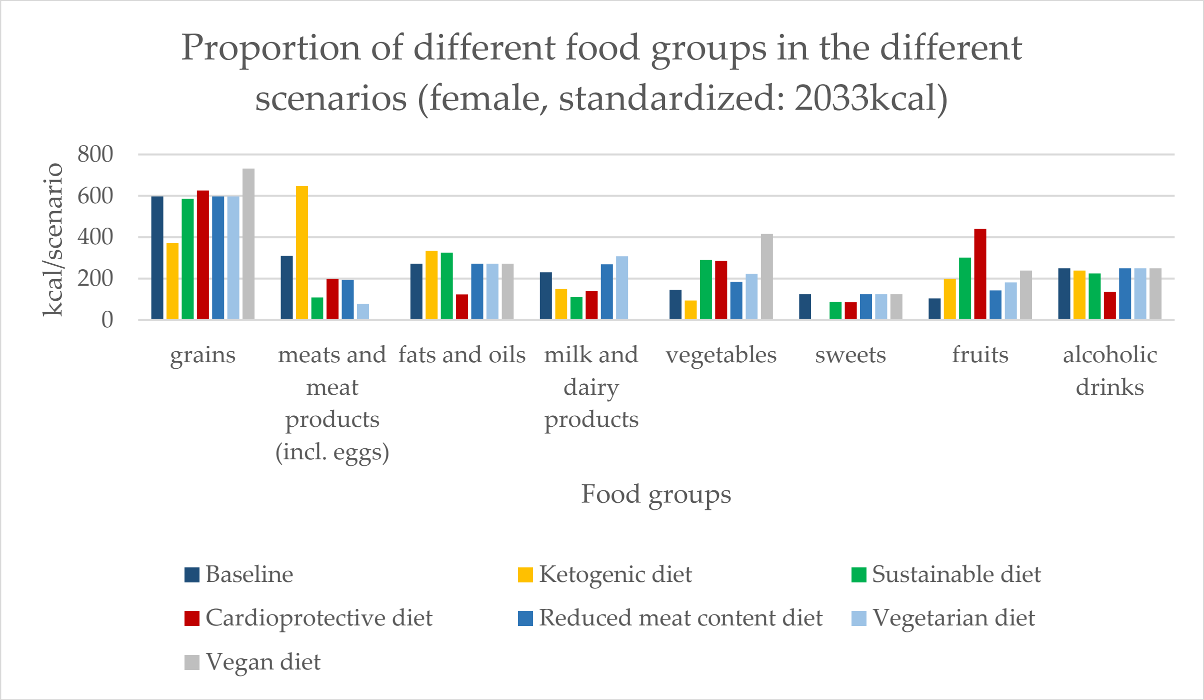

Supplement 3

Detailed description of dietary quality scores and integrated dietary quality value

(1) Qualifying nutrients: nutrients that are considered to be "good". The population's intake level of them is either adequate or low and a reasonably higher intake level is not related to health-risks [1-5]. In other words, diets that are rich in these nutrients are beneficial. In the case of qualifying nutrients, the scores increase positively with the nutrient density value up to 150% of the dietary reference value. At 150%, the scores will not increase further, so extreme nutritional density values will not be "rewarded". In the case of nutrients included in this group, toxicity should be considered only at an extreme intake value which is not realistic [5].

N_Q = if $N_s < Nr*1.5$ true: N_s / N_r, false: 1.5

(2) Disqualifying nutrients: nutrients that are considered to be "bad". The population's intake level of them is high and related to health-risks [1-5]. In other words, diets that are rich in these nutrients are unhealthy. Similar studies often include disqualifying nutrients in their calculations [6, 7]. In the case of nutrients included in this group, "less is more", so scores will decrease in correlation with the increase of the nutritional density values above the recommended maximum. In the case of nutritional density values that are under the maximum recommended intake, scores will increase in correlation with the increase of the nutritional density value. The score value for sugar is based on a relative comparison; the reference intake level is the calculated intake of the population intake based on [1]. Even though there are recommendations for added sugar intake, calculations were mostly based on unprocessed food items, so instead of dietary reference values, the relative difference compared to the baseline scenario gave the score values for sugar.

$N_{DQ} = 1- (N_s / N_r-1)$

(3) Macronutrients with a recommended intake range: nutrients that contribute to energy intake. These usually have a dietary reference value that includes a relative range based on the total recommended energy intake or body weight. Total carbohydrates, total fat and total protein are classified in these groups. Even though dietary fibers, sugars, cholesterol and saturated fatty acids are categorized as types of macronutrients, they were classified in different subgroups since they have a differentiated role in human health [5]. Total fat was classified as a disqualifying nutrient in dietary quality score$_{HUN}$ since only a maximum dietary reference value was determined due to the high population intake level, and a lower intake would be beneficial [1]. However, in the summary report of the EFSA there is a recommended intake range, so in the case of dietary quality score$_{EFSA}$ it is classified as a macronutrient with a recommended intake range [5]. To calculate the exact dietary reference values for macronutrients (as they are within the range of the recommended energy intake percentage) it was necessary to calculate as if for a reference human being, so for both dietary quality score$_{HUN}$ and dietary quality score$_{EFSA}$ a theoretical human of average age, weight and physical activity level was considered (for details look for S4). In the case of nutrients falling into this group, there is a recommended range, so it is problematic to classify them as qualifying or disqualifying. Scores will increase in correlation with nutritional density values up to the maximum level of the recommended range. If the nutritional density values exceed the maximum level of the recommended range, the scores will decrease in correlation with the increase above the maximum value.

N_{range} = if $N_s < N_{rmax}$ true: N_s / N_{rave}, false: $1- (N_s / N_{rmax}-1)$

(4) Recommended intake ratio of two nutrients: nutrients that have an interaction with their absorption and/or utilization, and the recommendation for their relative intake proportions, is based on the publication of [2-4]. In the case of these nutrients, scores will decrease if the ratio changes to favour disadvantageous nutrients (Na and P) and will increase if the ratio changes to favour advantageous nutrients (K and Ca).

$N_{ratio} = 1 - (Na_S / K_S - 1), (Ca_S / P_S - 2) - 1$

The algorithm of the total score:

$DQS_{HUN} = (N_{Q1} + N_{Q2} \ldots + N_{QX}) + (N_{DQ1} + N_{DQ2} + \ldots N_{DQX}) + (N_{range1} + N_{rage2} + \ldots N_{rangeX}) + (N_{ratio1} + N_{ratio2} + \ldots N_{ratioX})$

$DQS_{EFSA} = (N_{Q1} + N_{Q2} \ldots + N_{QX}) + (N_{DQ1} + N_{DQ2} + \ldots N_{DQX}) + (N_{range1} + N_{rage2} + \ldots N_{rangeX}) + (N_{ratio1} + N_{ratio2} + \ldots N_{ratioX})$

The algorithm of the integrated dietary quality value (IDQV):

Integrated dietary quality score (IDQV): $[(DQS_{HUN}\ scenario_x / DQS_{HUN}\ scenario_{HDNSS\text{-}original} * 100) - 100 + (DQS_{EFSA}\ scenario_x / DQS_{HUN}\ scenario_{HDNSS\text{-}original} * 100) - 100] / 2$

where:

IDQV = Integrated dietary quality value

DQS = Dietary Quality Score

N_Q = Qualifying nutrient

N_{DQ} = Dis-qualifying nutrient

N_{range} = Nutrient with recommended intake range

N_{ratio} = Recommended intake ratio of two nutrients

N_s = Amount of the nutrient in the scenario

N_r = Recommended intake level of the nutrient

N_{rmax} = Maximum value of the recommended intake range of the nutrient

N_{rave} = Average value of the recommended intake range of the nutrient

Na_S = Amount of Na in the scenario

K_S = Amount of K in the scenario

Ca_S = Amount of Ca in the scenario

P_S = Amount of P in the scenario

References

1. Sarkadi Nagy, E.; Bakacs, M.; Illés, É.; Nagy, B.; Varga, A.; Kis, O.; Schreiberné Molnár, E.; Martos, É., Országos Táplálkozás és Tápláltsági Állapot Vizsgálat–OTÁP2014. II. A magyar lakosság energia-és makrotápanyag-bevitele. *Orv. Hetil.* **2017,** 158, (15), 587-597. https://doi.org/10.1556/650.2017.30718
2. Schreiberné Molnár, E.; Nagy-Lőrincz, Z.; Nagy, B.; Bakacs, M.; Kis, O.; Sarkadi Nagy, E.; Martos, É., Országos Táplálkozás-és Tápláltsági Állapot Vizsgálat-OTÁP2014. V. A magyar lakosság vitaminbevitele. *Orv. Hetil.* **2017,** 158, (33), 1303-1313.
3. Nagy, B.; Nagy-Lőrincz, Z.; Bakacs, M.; Illés, É.; Sarkadi Nagy, E.; Erdei, G.; Martos, É., Országos Táplálkozás és Tápláltsági Állapot Vizsgálat–OTÁP2014. IV. A magyar lakosság mikroelem-bevitele. *Orv. Hetil.* **2017b,** 158, (21), 803-810.
4. Nagy, B.; Nagy-Lőrincz, Z.; Bakacs, M.; Illés, É.; Sarkadi Nagy, E.; Martos, É., Országos Táplálkozás és Tápláltsági Állapot Vizsgálat–OTÁP2014. III. A magyar lakosság makroelem-bevitele. *Orv. Hetil.* **2017a,** 158, (17), 651-661.

5. European Food Safety Authority (EFSA) *Dietary Reference Values for nutrients Summary report EFSA Supporting Publications*; 2017; pp 1-98.
6. Masset, G.; Soler, L.-G.; Vieux, F.; Darmon, N., Identifying sustainable foods: the relationship between environmental impact, nutritional quality, and prices of foods representative of the French diet. *Journal of the Academy of Nutrition and Dietetics* **2014,** 114, (6), 862-869. https://doi.org/10.1016/j.jand.2014.02.002
7. Masset, G.; Vieux, F.; Verger, E. O.; Soler, L.-G.; Touazi, D.; Darmon, N., Reducing energy intake and energy density for a sustainable diet: a study based on self-selected diets in French adults. *The American journal of Clinical Nutrition* **2014,** 99, (6), 1460-1469. https://doi.org/10.3945/ajcn.113.077958

Supplement 4

	Age	Physical activity level	Recommended energy intake
**Reference male**			
Dietary quality score$_{HUN}$	average of age group 18-29 and 70+	moderately active	2400 kcal/day
Dietary quality score$_{EFSA}$	average of age group 19-29 and 70-79	moderately active (1.6* basic metabolic rate)	2472 kcal/day
**Reference female**			
Dietary quality score$_{HUN}$	average of age group 18-29 and 70+	moderately active	1850 kcal/day
Dietary quality score$_{EFSA}$	average of age group 19-29 and 70-79	moderately active (1.6* basic metabolic rate)	1994 kcal/day

Sources:[1, 2]

References:

1. Sarkadi Nagy, E.; Bakacs, M.; Illés, É.; Nagy, B.; Varga, A.; Kis, O.; Schreiberné Molnár, E.; Martos, É., Országos Táplálkozás és Tápláltsági Állapot Vizsgálat–OTÁP2014. II. A magyar lakosság energia-és makrotápanyag-bevitele. *Orv. Hetil.* **2017,** 158, (15), 587-597. https://doi.org/10.1556/650.2017.30718
2. European Food Safety Authority (EFSA) *Dietary Reference Values for nutrients Summary report EFSA Supporting Publications*; 2017; pp 1-98.

Supplement 5

Energy and macronutrients (values are calculated based on the reference humans (S4))					
	**Energy (kcal)**	**Dietary fiber (g)**	**Sugars (g)**	**Cholesterol (mg)**	**Saturated fatty acids (g)**
Male					
EFSA	2472	25	32	na	27
HUN	2400	25	32	300	19
Female					
EFSA	1994	25	32	na	22
HUN	1850	25	32	300	14

Dietary reference values included in the dietary quality scores

Macronutrients with recommended intake range (values are calculated based on the reference humans (S4))							
		**Total protein (g)**		**Total carbohydrate (g)**		**Total fat (g)**	
		HUN	EFSA	HUN	EFSA	HUN	EFSA
Male	min	60	62	330	278	76	55
	max	90	125	360	370	80	96
Female	min	46	50	254	224	61	44
	max	69	100	278	299	72	78

Water soluble vitamins							
	**Thiamin (mg/d)**	**Riboflavin (mg/d)**	**Niacin (NE)**	**B6 (mg/d)**	**Folate (µg/d)**	**B12 (µg/d)**	**C (mg/d)**
Male							
EFSA	1	1.6	16.6	1.7	330	na	110
HUN	1.1	1.6	18	1.3	200	2	90
Female							
EFSA	0.8	1.6	13.4	1.6	330	na	95
HUN	0.9	1.3	14	1.3	200	2	90

Fat soluble vitamins			
	**A (µg/d RE)**	**E (mg)**	**K (µg/d)**
Male			
EFSA	750	na	70
HUN	1000	15	na
Female			
EFSA	650	na	70
HUN	800	15	na

Minerals							
	**Calcium (mg/d)**	**Magnesium (mg/d)**	**Zinc (mg/d)**	**Phosphorus (mg/d)**	**Potassium (mg/d)**	**Iron (mg/d)**	**Sodium (mg)**
Male							
EFSA	950	350	16.3	550	3500	11	na
HUN	800	350	10	620	3500	10	2000
Female							
EFSA	950	300	12.7	550	3500	16	na
HUN	800	300	9	620	3500	15	2000

Mineral ratio		
	**Na:K**	**Ca:P**
Male		
HUN	1:1	2:1
Female		
HUN	1:1	2:1

Source: [1], [2], [3], [4], [5]

References:

1. Sarkadi Nagy, E.; Bakacs, M.; Illés, É.; Nagy, B.; Varga, A.; Kis, O.; Schreiberné Molnár, E.; Martos, É., Országos Táplálkozás és Tápláltsági Állapot Vizsgálat–OTÁP2014. II. A magyar lakosság energia-és makrotápanyag-bevitele. *Orv. Hetil.* **2017,** 158, (15), 587-597. https://doi.org/10.1556/650.2017.30718
2. Nagy, B.; Nagy-Lőrincz, Z.; Bakacs, M.; Illés, É.; Sarkadi Nagy, E.; Erdei, G.; Martos, É., Országos Táplálkozás és Tápláltsági Állapot Vizsgálat–OTÁP2014. IV. A magyar lakosság mikroelem-bevitele. *Orv. Hetil.* **2017b,** 158, (21), 803-810.
3. Nagy, B.; Nagy-Lőrincz, Z.; Bakacs, M.; Illés, É.; Sarkadi Nagy, E.; Martos, É., Országos Táplálkozás és Tápláltsági Állapot Vizsgálat–OTÁP2014. III. A magyar lakosság makroelem-bevitele. *Orv. Hetil.* **2017a,** 158, (17), 651-661.
4. Schreiberné Molnár, E.; Nagy-Lőrincz, Z.; Nagy, B.; Bakacs, M.; Kis, O.; Sarkadi Nagy, E.; Martos, É., Országos Táplálkozás-és Tápláltsági Állapot Vizsgálat-OTÁP2014. V. A magyar lakosság vitaminbevitele. *Orv. Hetil.* **2017,** 158, (33), 1303-1313.
5. European Food Safety Authority (EFSA) *Dietary Reference Values for nutrients Summary report EFSA Supporting Publications*; 2017; pp 1-98.
